# Factors Associated with Increased Neuroretinal Rim Thickness Measured Based on Bruch’s Membrane Opening-Minimum Rim Width after Trabeculectomy

**DOI:** 10.3390/jcm10163646

**Published:** 2021-08-18

**Authors:** Do-Young Park, Soon-Cheol Cha

**Affiliations:** Department of Ophthalmology, Yeungnam University Hospital, Yeungnam University College of Medicine, Daegu 42415, Korea; dypark@ynu.ac.kr

**Keywords:** neuroretinal rim reversal, Bruch’s membrane opening-minimum rim width, trabeculectomy, intraocular pressure

## Abstract

Purpose: To investigate the factors associated with an increase in the neuroretinal rim (NRR) thickness measured based on Bruch’s membrane opening-minimum rim width (BMO-MRW) after trabeculectomy in patients with primary open-angle glaucoma (POAG). Methods: We analyzed the BMO-MRW using spectral-domain optical coherence tomography (SD-OCT) of patients with POAG who underwent a trabeculectomy for uncontrolled intraocular pressure (IOP) despite maximal IOP reduction treatment. The BMO-MRW was measured before and after trabeculectomy in patients with POAG. Demographic and systemic factors, ocular factors, pre- and post-operative IOP, and visual field parameters were collected, together with SD-OCT measurements. A regression analysis was performed to investigate the factors that affected the change in the BMO-MRW after the trabeculectomy. Results: Forty-four eyes of 44 patients were included in the analysis. The IOP significantly decreased from a preoperative 27.0 mmHg to a postoperative 10.5 mmHg. The mean interval between the trabeculectomy and the date of post-operative SD-OCT measurement was 3.3 months. The global and sectoral BMO-MRW significantly increased after trabeculectomy, whereas the peripapillary retinal nerve fiber layer thickness did not show a difference between before and after the trabeculectomy. Younger age and a greater reduction in the IOP after the trabeculectomy were significantly associated with the increase in the BMO-MRW after trabeculectomy. Conclusions: The NRR thickness measured based on the BMO-MRW increased with decreasing IOP after trabeculectomy, and the increase in the BMO-MRW was associated with the young age of the patients and greater reduction in the IOP after trabeculectomy. Biomechanically, these suggest that the NRR comprises cells and substances that sensitively respond to changes in the IOP and age.

## 1. Introduction

Reducing intraocular pressure (IOP) from glaucoma filtration surgery is accompanied by dynamic structural changes in the optic nerve head (ONH) [1,2,3,4,5,6,7,8,9,10,11,12]. These changes are called “reversal of disc cupping” because neuroretinal rim (NRR) tissue thickening is noted in funduscopic imaging and confocal scanning laser ophthalmoscopy [4,5,9,10,12]. Since optical coherence tomography (OCT) became available, several studies have been conducted on the detailed structural changes in the ONH and peripapillary retinal nerve fiber layer (RNFL) thickness after glaucoma filtration surgery [1,2,3,7,8,11]. Studies using OCT have found that the lamina cribrosa (LC) depth decreased after trabeculectomy, whereas the peripapillary RNFL thickness was unchanged [2,3,7].

More recently, Bruch’s membrane opening (BMO)-related OCT parameters have enabled the quantification of the NRR tissue of the ONH [13,14]. As expected, the representative parameters—BMO-MRW and BMO-minimum rim area—were reported to increase after trabeculectomy [3,7,11,15].

The increase in the NRR thickness after trabeculectomy is believed to occur secondary to the relief of compressive and stretch forces on the NRR tissue due to a reduced IOP [3]. Depending on the characteristics of the cells or substances filling the NRR tissue, the degree of the increase in the NRR thickness after trabeculectomy might be different. However, it is not yet known which factors affect the increase in NRR thickness following a postoperative IOP decrease.

In this study, using spectral-domain OCT (SD-OCT) and analyzing the BMO-based parameters of the NRR before and after trabeculectomy, we aimed to determine the factors that affect the changes in NRR thickness due to an IOP decrease after trabeculectomy.

## 2. Methods

### 2.1. Participants

For this retrospective, interventional study, all patients who underwent trabeculectomy at Yeungnam University Hospital from March 2020 to December 2020 were reviewed, and patients who met the following inclusion and exclusion criteria were selected. The study was approved by the Yeungnam University Hospital Institutional Review Board (IRB) and followed the tenets of the Declaration of Helsinki (IRB no. 2021-03-066). Considering the nature of the retrospective study, the requirement to obtain informed consent was waived by the IRB of the Yeungnam University Hospital.

The inclusion criteria were as follows: (1) patients aged 20 years or older who were diagnosed with primary open-angle glaucoma (POAG) and underwent trabeculectomy for uncontrolled IOP despite the maximum IOP reduction treatment, (2) patients who underwent at least one disc SD-OCT examination within 1 month prior to the glaucoma surgery, (3) patients who underwent at least one disc SD-OCT examination between 1 and 6 months after glaucoma surgery, and (4) patients whose IOP remained stable below 20 mmHg from immediately after the surgery to the time of the postoperative SD-OCT scan. POAG was diagnosed based on the following criteria: (1) the presence of typical glaucomatous optic disc changes (increased cupping, focal or diffuse loss of the NRR, or an RNFL defect), (2) glaucomatous visual field (VF) defect in at least two consecutive tests, (3) an open angle observed during a gonioscopic examination, and (4) IOP > 21 mmHg with or without the use of an antiglaucomatous medication. All patients underwent limbal- or fornix-based trabeculectomy with mitomycin C, which was performed by a glaucoma specialist (DYP, CSC).

Regarding the exclusion criteria, patients who underwent glaucoma surgery with a diagnosis of angle-closure glaucoma or secondary glaucoma, such as neovascular glaucoma, uveitic glaucoma, or exfoliation glaucoma, were excluded because factors such as sudden fluctuations in the IOP before surgery, ONH ischemia, or ocular inflammation may affect the analysis of changes in the ONH according to the decrease in the IOP after surgery. Cases of a needling procedure or additional surgery due to an increased IOP after surgery, cases requiring additional procedures because of hypotony, or cases with an IOP of less than 6 mmHg were excluded.

The following parameters were collected from the included patients: age, sex, presence of hypertension or diabetes, phakic status, average IOP after maximum medical IOP-lowering treatment within 3 months before surgery, average IOP within 6 months after surgery, axial length, central corneal thickness (CCT), and VF parameters (mean deviation (MD), pattern standard deviation, and VF index). SD-OCT measurements (BMO and RNFL parameters) taken before and after surgery were also collected, together with the period from the surgery to the SD-OCT measurement.

### 2.2. Measurement of Bruch’s Membrane Opening-Minimum Rim Width Using Spectral-Domain Optical Coherence Tomography

A Spectralis SD-OCT (Heidelberg Engineering GmbH, Heidelberg, Germany) was used to measure BMO-related parameters in the ONH. SD-OCT imaging was performed in accordance with the standard operating procedures. The scanning was focused on the center of the BMO, and 24 radial equidistant cross-sectional images were obtained. The BMO-MRW and RNFL thickness values were automatically calculated on a global and sectoral basis using the device’s standard operating software. The BMO area was also calculated automatically. If necessary, automated delineation of the internal limiting membrane and BMO were manually corrected.

### 2.3. Statistical Analyses

A paired *t*-test or a Wilcoxon signed-rank test was performed depending on data normality to compare the variables before and after trabeculectomy. We performed univariable and multivariable regression analyses using a generalized linear model to identify factors associated with an increase in the BMO-MRW after trabeculectomy. Characteristics with a *p*-value < 0.1 in the univariable analysis were included in the multivariable analysis. In the multivariable regression analysis, two models were used to avoid multicollinearity. Beta coefficients were calculated based on a 10 µm increase in the BMO-MRW. A *p*-value < 0.05 was considered statistically significant. Statistical analyses were performed using IBM SPSS software version 24.0 (IBM Corp., Armonk, NY, USA) and R statistical package version 3.5.3 (R Foundation for Statistical Computing, Vienna, Austria).

## 3. Results

Sixty-six eyes of 66 patients with POAG underwent trabeculectomy from March 2020 to December 2020. Of these, 10 and 6 eyes were excluded from the analysis due to the absence of an OCT examination before and after surgery, respectively. Four eyes were excluded due to the presence of postoperative hypotony. Two eyes were excluded due to poor OCT image quality. Finally, 44 eyes of 44 patients were included in the analysis.

Demographic and baseline information of the patients is summarized in Table 1. The mean age of the 44 patients was 62.5 years (standard deviation (SD), 15.1 years), of whom 33 (75%) were male. The mean interval between trabeculectomy and the postoperative SD-OCT examination was 3.3 months (SD, 1.9 months).

The IOP significantly decreased from a preoperative 27.0 mmHg (SD, 6.6 mmHg) to a postoperative 10.5 mmHg (SD, 3.3 mmHg). The global BMO-MRW significantly increased from a preoperative value of 151.9 μm to a postoperative value of 181.8 μm (*p* < 0.001). In addition to the global BMO-MRW, the BMO-MRW by sector also significantly increased postoperatively, except for the inferotemporal (TI) sector (Table 2). The BMO area and average peripapillary RNFL thickness did not show a significant difference between preoperatively and postoperatively (Table 2).

**Figure 1:**
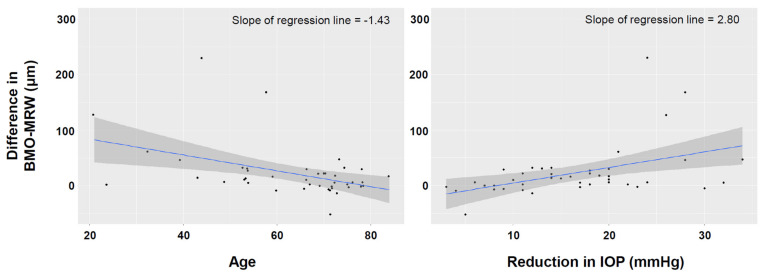
Scatter plot showing the relationship between the increase in the Bruch’s membrane opening-minimum rim width (BMO-MRW) after trabeculectomy and the patients’ age (left) and the amount of IOP reduction after trabeculectomy (right).

Univariable regression analysis found that the increase in the global BMO-MRW after trabeculectomy was greater when the patient’s age was lower, the CCT was thinner, the VF MD was better, the preoperative average RNFL thickness was greater, and the IOP reduction was higher (Table 3). Multivariable regression analyses found that younger age and greater reduction in the IOP after trabeculectomy remained significant factors that affected the increase in the BMO-MRW after trabeculectomy (Table 3, Figure 1). Unlike the BMO-MRW, the change in the average RNFL thickness was not affected by the patients’ age or the amount of reduction in IOP after trabeculectomy (Supplementary Table S1).

## 4. Discussion

NRR thinning is a major change that is observed in the disc in glaucoma, and it is important in diagnosing glaucoma and judging the progression of glaucoma. NRR thinning is known to be dynamically reversed after glaucoma surgery [2,3,4,5,6,7,8,9,10,11,12]. Therefore, understanding the factors that are associated with these changes can help to determine the progression of patients with glaucoma after glaucoma surgery. In addition, identifying the factors that are associated with the reversal of NRR thinning has important implications in that it may help to better understand the biomechanical properties of the NRR and infer the pathogenesis of disc cupping. In this study, we found that the BMO-MRW increased significantly after trabeculectomy, whereas there was no significant change in the RNFL. In multivariable regression analysis, changes in BMO-MRW were greater with a greater reduction in the IOP after trabeculectomy, even more so in younger patients.

Several studies reported changes in the structure of the ONH after glaucoma filtration surgery. Previously, it was reported that the NRR became thicker on two-dimensional fundus photographs [4,5,6,9,10,12]. Subsequently, through cross-sectional ONH analysis using SD-OCT, it was found that the depth of the LC decreased and its thickness increased [8]. Changes in the LC depth after the IOP reduction via glaucoma filtration surgery were also reported to be observed in myopic eyes with a tilted disc [16]. It was also reported that the LC curvature flattened and the LC depth decreased as the IOP decreased after surgery [17,18]. These changes were thought to be the result of connective tissue remodeling and collagen rearrangement occurring in the LC. Previous studies showed that the amount of change in the LC depth or curvature after glaucoma filtration surgery was related to the higher perioperative IOP reduction and younger patients’ age [8,17], which were also found in this study to be associated with changes in the BMO-MRW after surgery. These suggest that, similar to the LC, the NRR measured based on the BMO-MRW had a composition that can be reversibly changed following lowering of IOP by filtering surgery, which could be affected by the amount of IOP reduction and young age.

Histologically, it is known that the composition of the NRR and LC differs [19]. In other words, when the nerve fiber axon passes through the NRR-containing prelaminar and LC, the cells and substances that consist of the surrounding structures are changed. In the peripapillary area, thin-bodied glial cells are distributed parallel to the axons [19,20]. In the NRR-containing prelamina, glial cells show a loose arrangement and glial fibers are distributed perpendicular to the nerve fiber bundle [19,21]. In the LC, connective tissue consisting of collagen, elastin, and laminin separates the glial cells and axons as the main components. Different from the LC, the NRR is known to have little connective tissue. Considering these characteristics, the structural changes in the NRR according to the fluctuation in the IOP after surgery might be related to the plasticity of distribution of the glial cells. In fact, in an animal model of glaucoma, it was reported that as the IOP increased, the processes of astrocytes thickened and the distribution of the astrocytes changed from perpendicular to parallel to the axons [22,23]. In addition, there was a study that showed that the population of glial cells in the optic nerve in rodents varies with age [24,25]. Therefore, we assumed that age-dependent changes that occur in glial cells when the IOP increases might be reversed when the IOP decreases after trabeculectomy.

Unlike the BMO-MRW, in this study, the peripapillary RNFL did not show a reversible change according to the postoperative IOP reduction, which is consistent with the results of previous studies [2,3,7]. This implies that it was not the thickness or number of axons, but rather the properties and distribution of glial cells surrounding the axons that were different in the peripapillary RNFL and BMO-MRW according to the decrease in the IOP after trabeculectomy. More studies assessing the characteristics and distributions of the glial cells comprising the ONH in humans or primates, their changes with age and IOP, and plasticity and reversibility of the changes are required.

This study has several limitations. First, in this study, we did not evaluate the change in LC-related parameters after trabeculectomy, such as the LC depth, thickness, or curvature. This was because we analyzed the ONH only with radial scan, which is not appropriate for analyzing the LC. Therefore, we were not able to obtain the results regarding the change in the LC in response to an IOP decrease after trabeculectomy, and it was not possible to interpret the change in the BMO-MRW after surgery in relation to the change in the LC. Further studies assessing the correlation between the changes in the BMO-MRW after trabeculectomy and changes in the LC and whether the factors associated with these changes have an independent effect are needed. Second, we did not observe long-term changes in the BMO-MRW after trabeculectomy. Previously, it was reported that after 6 months of glaucoma drainage device surgery or trabeculectomy, the BMO-MRW was not significantly different from preoperative values [7,26]. Therefore, our results should be interpreted as showing that the transient increase in the BMO-MRW following postoperative IOP decrease was associated with age and the amount of IOP reduction after trabeculectomy. It is not yet known whether age and amount of IOP reduction are also related to the duration for the BMO-MRW to normalize after surgery. Third, this study was not conducted in a prospective manner; thus, we cannot exclude the potential selection bias. However, most of the parameters analyzed in this study, including OCT parameters, were items that are examined in a routine manner in our clinic before surgery and within 6 months after surgery. A prospective study with regular follow-up OCT examinations after trabeculectomy is needed in the future.

In conclusion, by quantitatively analyzing the BMO-MRW measured using SD-OCT, we confirmed that the NRR thickness increased after trabeculectomy. The increase in the BMO-MRW was related to the younger age of the patients and a greater reduction in the IOP after trabeculectomy. Clinically, we should consider these when judging the progression of glaucoma based on changes in the NRR after trabeculectomy. Biomechanically, these suggest that the cells or substances that make up the NRR respond to changes in the IOP after trabeculectomy, which may vary depending on age and the IOP.

## Figures and Tables

**Table 1:** Demographic and baseline characteristics of the patients.

Variables	Total Subjects (*n* = 44)
Age, years	62.5 ± 15.1
Gender, male/female	33/11
Diabetes, yes/no	15/29
Hypertension, yes/no	12/32
Phakia/pseudophakia	28/16
Central corneal thickness, μm	548.1 ± 37.4
Axial length, mm	24.2 ±1.3
Visual field MD, dB	−20 ± 7.5
Visual field PSD, dB	8.4 ± 3.0
Visual field VFI, %	44.7 ± 26.5
Average RNFL thickness, μm	62.5 ± 22.4
BMO-area, μm^2^	2.32 ± 0.56
Global BMO-MRW, μm	151.9 ± 45.5

IOP: intraocular pressure; MD: mean deviation; PSD: pattern standard deviation; dB: decibel; VFI: visual field index; RNFL: retinal nerve fiber layer; BMO-MRW: Bruch’s membrane opening-minimum rim width. Data are presented as mean ± standard deviation or *n* (frequency).

**Table 2:** Intraocular pressure, BMO-MRW, and RNFL thickness at baseline and the postoperative OCT examination.

	Preoperative	Postoperative	*p*-Value
IOP, mmHg	27 ± 6.6	10.5 ± 3.3	**<0.001**
BMO-area, μm^2^	2.32 ± 0.56	2.29 ± 0.57	0.52
Global BMO-MRW, μm	151.9 ± 45.5	181.8 ± 84.7	**<0.001**
BMO-MRW_TS, μm	127.3 ± 55.1	143.1 ± 83	**0.007**
BMO-MRW_T, μm	132.4 ± 45	153.8 ± 75.7	**0.002**
BMO-MRW_TI, μm	138.1 ± 78.8	152 ± 108.1	0.06
BMO-MRW_NI, μm	189.3 ± 66.7	212.8 ± 94	**0.004**
BMO-MRW_N, μm	169.7 ± 55.7	195.6 ± 73.6	**<0.001**
BMO-MRW_NS, μm	161.4 ± 61.9	183.7 ± 70.5	**<0.001**
Average RNFL thickness, μm	62.5 ± 22.4	61.6 ± 19.7	0.68
RNFL_TS, μm	79.2 ± 42	71.6 ± 31.9	0.08
RNFL_T, μm	57.4 ± 25.8	57.1 ± 24.8	0.87
RNFL_TI, μm	71.6 ± 33.1	70.3 ± 37.1	0.72
RNFL_NI, μm	66.2 ± 28.2	67.8 ± 24.9	0.37
RNFL_N, μm	53.6 ± 23.6	54.9 ± 20.7	0.45
RNFL_NS, μm	70 ± 35.5	64.9 ± 28.4	0.20

IOP: intraocular pressure; BMO-MRW: Bruch’s membrane opening-minimum rim width; RNFL: retinal nerve fiber layer. TS: superotemporal; T: temporal; TI: inferotemporal; NI: inferonasal; N: nasal; NS: superonasal. Data are presented as mean ± standard deviation. Statistically significant *p*-values are shown in bold.

**Table 3:** Factors associated with an increase in the global BMO-MRW (per 10 µm) after trabeculectomy.

	Univariable		Multivariable							
			Model 1				Model 2			
Variables	Beta (95% CI)	*p*-Value	Beta (95% CI)			*p*-Value	Beta (95% CI)			*p*-Value
Age, per 1 year older	−0.19 (−0.31 to −0.08)	**0.001**	−0.11 (−0.20 to −0.02)			**0.02**	−0.10 (−0.19 to −0.00)			0.05
Gender, male	−0.96 (−5.38 to 3.46)	0.67								
Diabetes	−1.01 (−5.06 to 3.04)	0.63								
Hypertension	−0.77 (−5.20 to 3.65)	0.73								
Phakia/pseudophakia	−1.80 (−5.76 to 2.16)	0.38								
Duration, months	0.36 (−0.69 to 1.41)	0.51								
Central corneal thickness, 40 μm	−2.04 (−3.98 to −0.09)	**0.04**	−0.55 (−1.97 to 0.86)			0.45	−0.40 (−1.85 to 1.05)			0.59
Axial length, mm	0.78 (−1.31 to 2.87)	0.47								
Visual field MD, dB	0.28 (0.04 to 0.52)	**0.03**					0.09 (−0.09 to 0.28)			0.33
Visual field PSD, dB	0.25 (−0.39 to 0.89)	0.44								
Preoperative average RNFL thickness, 10 μm	0.80 (−0.04 to 1.63)	0.07	0.26 (−0.32 to 0.85)			0.38				
Preoperative global BMO-MRW thickness, 10 μm	0.28 (−0.14 to 0.70)	0.21								
Preoperative IOP, mmHg	0.13 (−0.16 to 0.43)	0.39								
Postoperative IOP, mmHg	−0.63 (−1.03 to −0.24)	**0.003**								
Reduction of IOP, mmHg	0.28 (0.11 to 0.45)	**0.002**	0.19 (0.01 to 0.37)			**0.04**	0.21 (0.04 to 0.38)			**0.02**

IOP: intraocular pressure; MD: mean deviation; PSD: pattern standard deviation; dB: decibel; RNFL: retinal nerve fiber layer; BMO-MRW: Bruch’s membrane opening-minimum rim width. Visual field MD and the average preoperative RNFL thickness were strongly associated with each other; thus, each was analyzed separately in the multivariable analysis. Beta coefficients were calculated based on the 10 µm increase in the BMO-MRW. Statistically significant *p*-values are shown in bold.

## Data Availability

The data presented in this study are available from the authors upon reasonable request. The data are not publicly available due to privacy and ethical issue.

## Supplementary Materials

The following are available online at https://www.mdpi.com/article/10.3390/jcm10163646/s1, Table S1: Factors associated with increase in the average RNFL thickness (per 10 µm) after trabeculectomy.
