# Supplementary material for: Factors Associated with Increased Neuroretinal Rim Thickness Measured Based on Bruch’s Membrane Opening-Minimum Rim Width after Trabeculectomy
Source: J Clin Med. 2021 Aug 18;10(16):3646. doi: 10.3390/jcm10163646 (PMC8397154; doi:10.3390/jcm10163646)
Supplement: Supplementary file 1 [file jcm-10-03646-s001.zip › jcm-1310319-supplementary.pdf]

Supplementary Table S1. Factors associated with an increase in the average RNFL thickness (per 10 μm) after trabeculectomy.

	Univariate		Multivariate	
Variables	Beta (95% CI)	*p*-Value	Beta (95% CI)	*p*-Value
Age, per 10-year older	−0.01 (−0.20 to 0.20)	0.89		
Gender, male	0.31 (−0.47 to 1.10)	0.43		
Diabetes	0.38 (−0.34 to 1.11)	0.31		
Hypertension	0.60 (−0.17 to 1.37)	0.13		
Phakia/pseudophakia	−0.16 (−0.87 to 0.55)	0.66		
Duration, months	−0.22 (−0.40 to -0.05)	**0.02**	−0.18 (−0.34 to −0.02)	**0.04**
Central corneal thickness, 40 μm	0.30 (−0.06 to 0.64)	0.11		
Axial length, mm	−0.16 (−0.43 to 0.10)	0.22		
Visual field MD, dB	−0.02 (−0.06 to 0.03)	0.48		
Visual field PSD, dB	−0.06 (−0.17 to 0.05)	0.29		
Preoperative average RNFL thickness, 10 μm	−0.24 (−0.38 to −0.10)	**0.001**	−0.21 (−0.35 to −0.08)	**0.003**
Preoperative global BMO-MRW thickness, 10 μm	−0.24 (−0.10 to 0.05)	0.52		
Preoperative IOP, mmHg	0.05 (0.00 to 0.10)	**0.05**	0.03 (−0.02 to 0.07)	0.26
Postoperative IOP, mmHg	−0.01 (−0.10 to 0.08)	0.77		
Reduction of IOP, mmHg	0.03 (−0.01 to 0.07)	0.13		

IOP: intraocular pressure; MD: mean deviation; PSD: pattern standard deviation; dB: decibel; RNFL: retinal nerve fiber layer; BMO-MRW: Bruch's membrane opening minimum rim width; Beta coefficients were calculated based on the 10 μm increase in the average RNFL thickness; Statistically significant *p*-values are shown in bold.
